# Acupuncture and related therapies for stress urinary incontinence: A protocol for systematic review and network meta-analysis

**DOI:** 10.1097/MD.0000000000021033

**Published:** 2020-07-10

**Authors:** Jiao Yang, Ying Cheng, Ling Zhao, Jiao Chen, Qianhua Zheng, Yaoguang Guo, Fanrong Liang

**Affiliations:** a Chengdu University of Traditional Chinese Medicine; b Hospital of Chengdu University of Traditional Chinese Medicine, Chengdu, China.

**Keywords:** acupuncture, network meta-analysis, stress urinary incontinence, systematic review

## Abstract

**Background:**

Stress urinary incontinence (SUI) is the most common type of urinary incontinence, affecting patients’ quality of life and sexual function. Lots of Clinical trials suggested that acupuncture is beneficial for SUI and various acupuncture methods have been widely used in clinic. However, the comparative efficacy and safety of these acupuncture methods remains unclear. Clinicians are confused to select the optimal way to treat SUI. This review aims to gather solid evidence in order to provide reliable reference in establishing guidelines for acupuncture treatment of SUI.

**Methods:**

Relevant databases including MEDLINE, Cochrane Library, EMBASE, Chinese National Knowledge Infrastructure, Chinese Biomedical Literature Database, Wanfang Database, the Chongqing VIP Chinese Science and Technology Periodical Database will be retrieved from their inception to April 2020. The quality of the included studies will be evaluated by the risk of bias tool and the evidence will be evaluated by Grading of Recommendations Assessment, Development and Evaluation System. Network meta-analysis will be conducted by using software R3.5.1. The primary outcome is the number of patients with self-reported continence and number of patients with self-reported improvement in SUI.

**Results:**

The results of this network meta-analysis will be submitted to a peer-reviewed journal for publication.

**Conclusion:**

the results may be useful for patients, clinicians, and guideline-makers to choose the optimal acupuncture method for SUI treatment.

## 1 Introduction

Stress urinary incontinence (SUI), the most common type of urinary incontinence, is defined as the involuntary loss of urine through physical exertion or effort, coughing or sneezing.^[1]^ Prevalence of this disease is different in various parts of the world. A study conducted in USA reported that an estimated 49.6% of adult women are affected by SUI.^[2]^ In China, the prevalence is14% in adult women.^[3]^ SUI has a social, psychological, physical and financial impact on life. Over 80% of women without accept any treatment, and less than 1% undergo surgical management.^[4]^ Because of the fear of leakage, patients’ quality of life and sexual function are often substantially impaired.^[5]^ SUI can severely impact the ability to carry out daily activities, resulting in embarrassment, insomnia and social isolation.^[6]^ Patients with SUI may be less likely to take part in physical activity, which in turn has a harmful impact on overall health because inactivity is a risk factor for many diseases.^[7]^ Furthermore, SUI can cause a considerable economic burden for patients and healthcare providers.^[8]^

Treatment methods for SUI includes behavioral therapy, medications, physiotherapy, devices, and surgery.^[9–12]^ Duloxetine is the recommended drug for the treatment of SUI, but it is poorly tolerated due to adverse effects or lack of efficacy. This result in high discontinuation rates.^[13]^ Pelvic floor muscle training (PFMT) is one of the conservative therapies for SUI, it is more effective than no treatment, placebo drug or inactive control treatments for women with SUI.^[14]^ However, Pelvic floor muscle training needs long-term adherence and its value remains uncertain for post-prostatectomy incontinence in men.^[15]^ If conservative treatment has not improved patients’ symptoms, surgery is usually suggested as a second-line option.^[16]^ But it has risks (such as urinary retention, impair the bladder or urethra and severe infection) that some people may find unacceptable.

Therefore, there is an urgent need for effective, lower cost, non-invasive treatment, especially for people living in low-income regions. As a minimally invasive treatment, acupuncture is reported to be effective in treating SUI.^[17–19]^ Although it has not yet known how acupuncture produces its effects (e.g., whether on blood, muscles, nerves, or energy), it is possible that it could desensitize the bladder through inhibition of capsaicin-sensitive C-fiber activation. In recent years, various acupuncture methods has been used in treating SUI,^[18–21]^ most of the trials only compare acupuncture methods with medicine or sham acupuncture methods and there are hardly any studies directly comparing difference acupuncture methods. Furthermore, whether a combination of multiple acupuncture methods or acupuncture methods plus other therapy is superior to single acupuncture is still unclear. Therefore, determining the best acupuncture methods for treating SUI is intractable.

This protocol will evaluate and rank the different acupuncture treatments by using the network meta-analysis (NMA) to analyze the direct and indirect randomized data,^[15,22]^ and it will provide evidence to guide the best practice in acupuncture for SUI.

## 2 Methods

This protocol has been registered on the international prospective register of systematic review (PROSPERO), and it was drafted according to the Preferred Reporting Items for Systematic Reviews and Meta-Analyses Protocols (PRISMA-P).^[23]^ The final results will be reported according to the recommendation from The Preferred Reporting Items for Systematic Reviews and Meta-Analyses Extension Statement for Reporting of Systematic Reviews Incorporating Network Meta-analyses.^[24]^

### 2.1 Criteria for considering studies for this review

#### 2.1.1 Types of studies

We will include randomized controlled trials (RCTs) reporting in English or Chinese without any regional restrictions. The first period of randomized cross-over trials will be also included. We will exclude Non-RCTs reviews, animal experimental studies, case report, expert experience, conference article and duplicated publications.

#### 2.1.2 Types of participants

Participants diagnosed with SUI will be included, regardless of age, race, duration of disease, weight, mode of delivery, or education.

#### 2.1.3 Types of interventions

We will define acupuncture as acupoint-based therapies^[25]^ (e.g., moxibustion, catgut embedding, electro-acupuncture, transcutaneous electrical acupoint stimulation, auricular acupuncture, scalp acupuncture, warm needling, manual acupuncture, acupoint injection, medium-frequency electric stimulation, and so on), regardless of needling techniques and stimulation method. We will rule out interventions without stimulating the acupoint.

#### 2.1.4 Types of control groups

Treatments in the comparison groups can be sham-acupuncture, placebo, pharmacotherapy or rehabilitation exercise therapy. Studies compared different type of acupuncture methods will be included

#### 2.1.5 Types of outcome measures

##### 2.1.5.1 Primary outcomes

(1) Cure: number of patients with self-reported continence.
(2) Improvement: number of patients with self-reported improvement in SUI (cured or improved).

##### 2.1.5.2 Secondary outcomes

The secondary outcomes include the following items:

1. Incontinence-specific quality-of-life (QoL) measures defined by authors or by any validated measurement scales such as International Consultation on Incontinence Questionnaire
2. QoL measures of general health status, for example, SF-36.
3. Quantification of symptoms (e.g., number of incontinence episodes, number of micturitions, pad tests).
4. Socioeconomic measures (e.g., costs of interventions, cost effectiveness of interventions in terms of incremental cost-effectiveness ratios, costs per quality-adjusted life year or cost-benefit ratios).
5. Adverse effects (e.g., skin or tissue damage, pain or discomfort, vascular, visceral or nerve injury, voiding dysfunction).
6. Residual urine volume
7. Pelvic floor muscle strength or ability to contract the pelvic floor muscles, or both.

### 2.2 Search methods for identification of studies

#### 2.2.1 Electronic searches

We will search the following databases from their inception to April 2020: MEDLINE, Cochrane Library, EMBASE, Chinese National Knowledge Infrastructure (CNKI), Chinese Biomedical Literature Database (CBM), Wanfang Database, the Chongqing VIP Chinese Science and Technology Periodical Database (VIP). we will also search grey literature from World Health Organization Clinical Trials Registry, ClinicalTrials.gov and Chinese clinical registry. Reference lists of articles will be retrieved as additional studies.

The following search headings (MeSH) will be used: “stress urinary incontinence”, “urinary stress incontinence,” urinary stress,” urinary”, “acupuncture,” “electro acupuncture,” “auriculotherapy,” “acupoint,” “needle,” “acupoint catgut embedding,” “moxibustion,” “transcutaneous electrical acupoint stimulation,” acupoint injection,” “randomized controlled trial,” randomized controlled,” “randomized, controlled,” “clinical trial.” Chinese translations of these search terms will be used for the Chinese databases. The search strategy for MEDLINE is shown in Table 1.

**Table 1:**
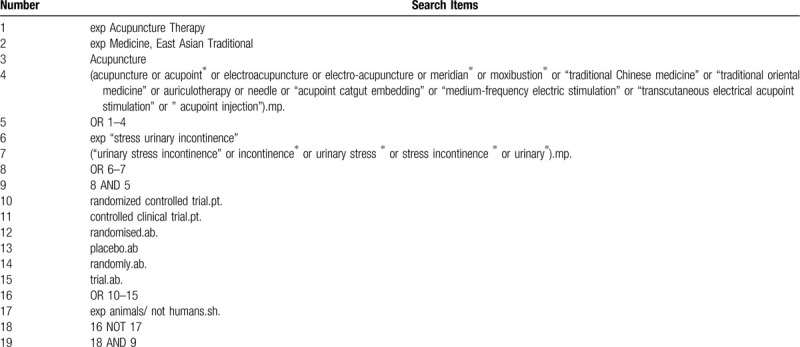
Illustration of the search strategies applied to this review (Table 1 Search strategy in Medline (Ovid SP)).

### 2.3 Data collection and analysis

#### 2.3.1 Selection of studies

**Figure 1:**
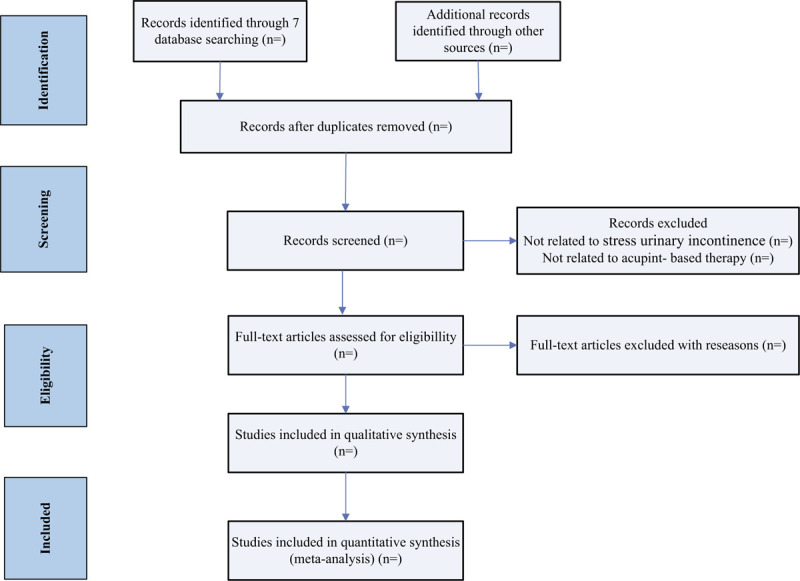
Illustration of the flow diagram of studies identified.

Two reviewers will independently search articles from titles and abstracts. Full texts will be searched for further evaluation when necessary. Then, the reviewers will examine the full text articles according to the inclusion criteria. For each excluded study, reason (s) for exclusion will be given. In cases of conflicting opinions, a third reviewer will be consulted to resolve any disagreement. Details of the selection process will be presented in the PRISMA flow chart (Fig. 1).

#### 2.3.2 Data extraction and management

Two reviewers will independently extract parameters from applicable studies including identification information (publication year and first author), general information (country, study type, number of centers, sample size), participants (age, sex, weight, mode of delivery, original disease), interventions (type of acupuncture, frequency/session/duration), comparator (if there is any, details of the treatment including name, dosage, frequency and course), outcomes (data and time points for each measurement, safety).

#### 2.3.3 Dealing with missing data

If the required data are ambiguous or not reported in the included articles, the corresponding authors of the studies will be contacted by telephone or email.

#### 2.3.4 Assessment of quality in included studies

The quality of the studies will be assessed according to the Cochrane risk of bias assessment tool.^[26]^ The main contents include: sequence generation, allocation concealment, blinding (or masks), incomplete data assessment, selective outcome reporting and other sources of bias. Then, the risk of bias for included studies will be classified as “low”, “unclear” and “high” risk of bias. The above content evaluation will be performed by 2 researchers, and any differences will be resolved through discussions or consultation with the third reviewer. The Grading of Recommendations Assessment, Development, and Evaluation (GRADE) system will be used to grading the quality of the evidence for main outcomes.^[27]^ Evidence quality will be graded as “high”, “moderate”, “low” or “very low” according to the GRADE rating standards.

### 2.4 Statistical analysis

#### 2.4.1 Pairwise meta-analysis

Traditional pairwise meta-analysis will be performed to compare treatments with direct evidence. Continuous outcomes will be calculated as standardized mean differences (SMDs) with 95% confidence interval (95% CI), and dichotomous outcomes will be calculated as OR with 95% CI. The heterogeneity of each pairwise comparison will be tested by I^2^ test. If I^2^ < 50%, fixed-effect model will be used, whereas a random-effect model will be used. And we will explore the reasons for the existence of heterogeneity from various aspects such as age, duration of disease and mode of delivery. If it is necessary, sensitivity analysis or meta-regression and subgroup analysis will be used to explore possible sources of heterogeneity. If 8 or more studies are involved in the meta-analysis, the publication bias will be evaluated using funnel plots. The funnel plot asymmetry will be evaluated by Egger test.

#### 2.4.2 Network meta-analysis

R3.5.1 software (AT&T, USA) will be used to perform NMA to synthesize direct and indirect evidence with Bayesian method.^[28]^ Reliability of the result of network meta-analysis largely depends on transitivity of the evidence network. However, it is difficult to determine transitivity by statistical analysis, so we will evaluate the transitivity from clinical and methodological variables that could act as effect modifiers across treatment comparisons.^[29]^ Moreover, the node splitting method will be performed to estimate the consistency of direct and indirect evidence in each closed loop according to the resultant *P*-value.^[30]^ Values of *P* > .05 indicate good consistency, otherwise, all inconsistencies will be reported (*P* < .05). The contribution of different designs to the final effect size estimated by the network meta-analysis will be evaluated by using net-heat plots.

The different acupuncture methods will be ranked by using P-score that measures the extent of certainty that a treatment is better than a control.^[31]^ 100% of the P-score indicates a treatment to be the best, while 0% of a P-score indicates a treatment to be the worst.

## 3 Discussion

Despite the lack of robust evidence, acupuncture is widely used for SUI in clinical practice. Choosing the optimal acupuncture method is difficult for clinicians due to the lack of comparative effect research. In this review, we will evaluate the comparative efficacy and safety of various acupuncture methods and combination regimens for the treatment of SUI. Network meta-analysis will summarize direct and indirect evidence aiming to provide a ranking of the acupuncture treatment for SUI.^[22,32]^ As less randomized trials directly evaluate the comparisons of different acupuncture methods, this study will provide the current best evidence by using network meta-analysis

This review will be the first to compare the efficacy and safety of various acupuncture therapies for SUI. But there are some limitations in this study. Frist, we will only include trials writing in Chinese or English. It could limit available data or result in language bias. Second, the quality of original trials will affect the quality of the pooled effects, so we will strictly control the quality of the included studies and the similarity of the basic characteristics of the study. Our review will offer credible evidence for the clinicians and encourage wider application of acupuncture for SUI.

## Acknowledgments

The authors thank Guixing Xu for her helpful assistance.

## Author contributions

**Data curation:** Jiao Yang and Ying Cheng.

**Methodology and investigation:** Jiao Chen and Qianhua Zheng.

**Resources:** Jiao Yang and Ling Zhao.

**Software:** Yaoguang Guo.

**Writing – original draft:** Jiao Yang and Yaoguang Guo.

**Writing – review & editing:** Fanrong Liang.
